# Bone Health Following Lifestyle‐Induced Weight Loss in Individuals With Overweight/Obesity: A Narrative Review

**DOI:** 10.1002/oby.70047

**Published:** 2025-10-10

**Authors:** Mélanie A. Legrand, Julien Paccou, Jean‐Michel Lecerf, Thierry Thomas, Roland Chapurlat, Bernard Cortet, Emmanuel Biver, Maria Papageorgiou

**Affiliations:** ^1^ Department of Rheumatology Edouard Herriot University Hospital, Inserm UMR 1033, Université de Lyon Lyon France; ^2^ Department of Rheumatology Université de Lille, CHU Lille Lille France; ^3^ Department of Nutrition and Physical Activity Institut Pasteur de Lille Lille France; ^4^ Department of Rheumatology Hôpital Nord, CHU Saint‐Etienne, Inserm U1059, Lyon University Saint‐Etienne France; ^5^ Division of Bone Diseases Geneva University Hospitals and Faculty of Medicine, University of Geneva Geneva Switzerland

**Keywords:** bone health, caloric restriction, exercise, fractures, overweight/obesity, weight loss

## Abstract

Although weight loss has many health benefits for people with overweight/obesity, its potential negative impact on bone health needs to be considered. This review provides a comprehensive overview of the effects of intentional weight loss achieved by lifestyle changes on bone health outcomes in adults with overweight/obesity and discusses potential mechanisms underlying the observed skeletal effects and protective measures to preserve bone health in this context. Weight loss achieved through lifestyle modifications increases surrogate markers of bone resorption and small but persistent reductions in bone mineral density at clinically relevant sites (mainly at the level of the hip). Based on limited available data, weight loss achieved by lifestyle modifications may increase fragility fractures. Combating sedentary lifestyles and promoting exercise, particularly resistance exercise, adequate intakes of calcium (diets and/or supplements), vitamin D supplementation, and higher dietary protein intakes could attenuate but not fully prevent the increased bone turnover or bone loss often associated with intentional weight loss. Further research needs to explore the skeletal effects of pragmatic interventions that match clinical scenarios, verify if changes in bone macro‐ and/or microstructure translate to an increased fracture risk, and investigate novel/combined strategies to improve bone health due to weight loss.

**Study Importance**

- What is already known?
  ○ People with overweight/obesity may have poorer bone quality and structure, and they may face a higher risk of fractures, especially at sites less commonly affected by osteoporosis, such as the ankle, lower leg, and upper arm.
  ○ In this population, weight loss does not improve bone health but may instead worsen existing bone alterations.
- What does this review add?
  ○ Lifestyle‐induced weight loss (typically 5%–10%) is linked to increased bone resorption and modest but lasting reductions in BMD, particularly at the hip, and may increase fragility fractures especially if other risk factors are present.
  ○ Regular exercise, with an emphasis on resistance exercise, adequate calcium intake (through diet and/or supplementation), vitamin D supplementation, and higher dietary protein consumption may attenuate, though often it does not fully prevent, the bone loss commonly associated with weight reduction.
- How might these results change the direction of research or the focus of clinical practice?
  ○ Further research is needed to clarify the skeletal effects of repeated weight loss attempts, identify individuals most susceptible to bone loss, further investigate changes in bone microstructure and fracture risk, and explore novel or combined strategies to preserve bone health during weight loss.
  ○ Our work can inform health care providers to promote a more holistic approach to weight management that encompasses bone health and guide them on how to advise patients on safe weight loss strategies that minimize unfavorable skeletal effects.

## 1 Introduction

The prevalence of overweight/obesity has increased significantly in recent decades worldwide, with over 2.5 billion adults (43%) having these conditions [1]. It is also projected that more than half the global population will be living with overweight/obesity within the next 12 years if current trends persist and there are no significant improvements in prevention and treatment [2]. Obesity is associated with several comorbidities (e.g., diabetes, cardiovascular diseases, various types of cancer), while even a 5%–10% weight loss can confer significant improvement. Lifestyle interventions (e.g., weight loss diets, physical activity, behavioral therapy) remain the cornerstone of obesity management further supported by pharmacotherapy and bariatric metabolic surgery [3, 4].

Reflecting the obesity epidemic and the urgency for effective treatment, the effects of obesity and weight loss on every body system, including the skeletal system, have attracted scientific interest. People living with obesity have long been thought to be protected against osteoporosis because they present with a normal or a higher bone mass than that of individuals with a normal weight as a result of mechanical and endocrine adaptations to an increased body weight [5, 6, 7, 8, 9]. Nevertheless, more recent evidence suggests that they have impairments in the quality of the bone matrix and structure, with these effects largely attributed to chronic inflammation and hormonal disturbances linked to increased adiposity [7, 8, 9, 10]. People living with obesity may also be at an increased fracture risk, particularly at skeletal sites less commonly affected by osteoporosis including the ankle, lower leg, and proximal humerus [6, 9, 11, 12].

Paradoxically, weight loss does not improve but rather exacerbates bone health alterations in this population. Understanding the relationship between intentional weight loss and bone health is important as it can contribute to a more holistic approach to weight management emphasizing not just weight loss but overall health, including bone health. It can also guide health care providers on how to advise patients on safe weight loss strategies that minimize unfavorable impacts on bone health, but also inform relevant public health initiatives targeting obesity and related health issues.

As such, this review aims to provide a comprehensive overview of the effects of intentional weight loss achieved by lifestyle changes (e.g., diet, exercise) on outcomes of bone health, namely bone turnover markers (BTMs), bone mineral density (BMD), bone microstructure, and fracture risk in adults with overweight/obesity, discusses potential mechanisms underlying the observed skeletal effects, and presents the current evidence on the protective measures to preserve bone health in this context.

## 2 Methods

For the purposes of this narrative review, we conducted a literature search using the MEDLINE database up to December 2024. Relevant studies were selected using a combination of keywords for lifestyle‐induced weight loss (caloric restriction, weight loss, diet, hypocaloric diet, lifestyle modifications, exercise/physical activity/training/physical fitness, and obesity/overweight) and skeletal health outcomes (bone, bone turnover or remodeling, bone mineral density, osteoporosis, bone microstructure/microarchitecture, and fracture). Additional studies were identified through an extensive manual search of the bibliographic references in the original papers and reviews. We reviewed observational and interventional studies, with a focus on randomized controlled trials (RCT) and available meta‐analyses on this subject. Studies were excluded if they focused on involuntary weight loss, weight loss induced by obesity medications, or bariatric surgery, unless they included at least one study arm where weight loss was achieved through lifestyle modifications.

## 3 Results

### 3.1 The Effects of Diet‐Induced Weight Loss on BTMs and BMD

In this section, we focus on interventional studies and meta‐analyses that have assessed the effects of diet‐induced weight loss on BTMs and areal BMD (aBMD), as dietary changes are the most common intervention for weight loss in individuals with overweight/obesity and these bone parameters are typically reported in studies with interventional designs and follow‐up periods ranging from a few months to 2–3 years. Interventions combining caloric restrictions and exercise and their effects on bone health are discussed in subsequent sections.

#### 3.1.1 BTMs

We evaluated studies reporting changes in serum procollagen type 1 N‐terminal propeptide (PINP) and C‐terminal telopeptide of type I collagen (CTX) concentrations (Table 1) because these markers are recommended as the reference ones for bone formation and resorption, respectively [25].

**TABLE 1:** Bone turnover marker (BTMS; PINP and CTX) changes in lifestyle‐induced weight loss.

Study	Population	Results
Hinton et al. [13] Intervention: moderate CR (↓ by ~600 kcal/day) + aerobic exercise Duration: 4–6 months Calcium and vitamin D intakes: insufficient Protein intake: NA Weight loss: ~10% (−8.5 ± 0.3 kg)	*n* = 40 premenopausal women Age: 39 ± 1 years BMI: 33.1 ± 0.6 kg/m^2^ (25.8–42.5 kg/m^2^) T2D: NA	↗ CTX (+ 28% ± 4%)
Villalon et al. [14] Intervention: aerobic exercise Duration: 6 months Calcium and vitamin D intakes: NA Protein intake: NA Weight loss: < 10% (−3.9 ± 3.5 kg)	*n* = 21 postmenopausal women Age: 56.8 ± 5.4 years (50–70 years) BMI: 29.6 ± 4.0 kg/m^2^ T2D: 0	↗ CTX (+ 34% ± 54%)
Sukumar et al. [15] RCT; Intervention: moderate CR (by ~500–600 kcal/day) + 2 levels of protein intake with controlled calcium intake Duration: 12 months Calcium and vitamin D intakes: 1.2 g/day and 400 IU/day, respectively Protein intake: 86 (high protein HP) or 60 (normal protein NP) g/day Weight loss: ~10% (−7.0 ± 4.5 kg)	*n* = 47 postmenopausal women Age: 58.0 ± 4.4 years BMI: 32.1 ± 4.6 kg/m^2^ T2D: NA	In both groups: ↔ P1NP No significant differences in P1NP between the 2 groups with different levels of protein intake
Shah et al. [16] RCT; Intervention: caloric restriction (↓ by ~500–750 kcal/day) for Diet (D) and Diet + exercise (DE) groups ± supervised aerobic exercise and protein replacement therapy for Exercise (E) and DE groups Duration: 12 months (6 months weight loss and 6 months weight maintenance) Calcium and vitamin D intakes: ~1500 mg/day and ~1000 IU/day, respectively Protein intake: adequate protein intake Weight loss: ~10% at 6 months in both D and DE groups vs. no change (E and C)	*n* = 107 older adults Age: 70 ± 4 years (> 65 years) BMI: 37.2 ± 4.5 kg/m^2^ (> 30 kg/m^2^) T2D: NA – C group, *n* = 27 – D group, *n* = 26 – E group, *n* = 26 – DE group, *n* = 28	At 12 months C group: ↔ P1NP, ↔ CTX D group ↗ CTX (+ 31% ± 53%), ↗ P1NP (+9% ± 29%) E group ↘ CTX (−15% ± 28%), ↘ P1NP (−15% ± 25%) DE group ↔ P1NP, ↔ CTX Exercise therapy prevented the weight loss–induced ↗ in CTX
Uusi‐Rasi et al. [17] Intervention: caloric restriction and 1 group (*n* = 12) used VLED Duration: 3 months Calcium intake: adequate, 888 (271) mg/day Vitamin D intake: NA Protein intake: NA Weight loss: < 10% at 3 months; −4.3 ± 4.5 kg (−14.8 kg loss to +2.1 kg gain)	*n* = 37 healthy premenopausal women Age: 42 ± 7 years BMI: 35.2 ± 5.2 kg/m^2^ (> 30 kg/m^2^) T2D: 0	↔ CTX, ↔ P1NP Modest weight loss did not modify BTMs
Uusi‐Rasi et al. [18] Intervention: intensive caloric restriction (VLED) Duration: 3 months Calcium intake: adequate, 860 ± 206 mg/day Vitamin D intake: NA Protein intake: NA Weight loss: ~10% at 3 months; −9.8 ± 4.3 kg (−18.8 kg loss to +1.9 kg gain)	*n* = 62 premenopausal women Age: 40.2 ± 5.2 years (25–45 years) BMI: 35.2 ± 5.2 kg/m^2^ (> 30 kg/m^2^) T2D: 0	Large (15.5%) ↗↗ CTX~+70%, ↗ P1NP +18.2% (12.9%–45.8%) Medium (10.5%) ↗ CTX~+45%, ↗ P1NP +16.1% (2.2%–31.8%) Low (5.9%) ↗ CTX~+39%, ↔ P1NP BTMs ↗ during the weight loss period (VLED)
Armamento‐Villareal et al. [19] RCT; Intervention: intensive lifestyle interventions = CR (by ~500–750 kcal/day) + exercise Duration: 6 months Calcium and vitamin D intakes: ~1500 mg/day and ~1000 IU/day, respectively Protein intake: NA Weight loss: ~10% at 6 months	*n* = 160 older adults (57 women, 36%) Age: 70 ± 5 years BMI: 36.2 ± 5.1 kg/m^2^ (> 30 kg/m^2^) T2D: NA – Resistance group (R), *n* = 40 – Aerobic group (A), *n* = 40 – Combination group (R + A), *n* = 40 – Control group (C), *n* = 40	R group: ↔ CTX, ↔ P1NP A group: ↗ CTX +33%, ↗ P1NP +16% R + A group: ↗ CTX +11%, ↔ P1NP C group: ↔ CTX, ↔ P1NP Resistance and combined aerobic and resistance exercise were associated with less weight loss–induced ↗ in BTMs
Villareal et al. [20] RCT; Intervention: caloric restriction (reduced by ~500–750 kcal/day) + exercise (aerobic exercise and strength exercise) Duration: 12 months Calcium and vitamin D intakes: 1200–1500 mg/day and 1000 IU/day, respectively Protein intake: NA Weight loss: ~10% at 6 months + weight maintenance 6 months	*n* = 27 (18 women) older adults, Age: 70 ± 5 years BMI: 39 ± 5 kg/m^2^ (> 30 kg/m^2^) T2D: NA – Treatment group, *n* = 17 – Control group, *n* = 10	At 6 months Treatment group: ↗ CTX 101% ± 79% Control group: ↔ CTX 12% ± 35% CTX ↗ in the treatment group but not in the control group
Josse et al. [21] RCT; Intervention: CR (by ~500 kcal/day) + daily exercise (aerobic exercise and protein replacement therapy) with varied intakes of protein and dairy foods Duration: 4 months Calcium and vitamin D intakes: 1200–1500 mg/day and 1000 IU/day, respectively Protein intake: Adequqte protein and low dairy (APLD) group: 0‐1 dairy servings/day, 15% of their daily energy from nondairy sources of high‐quality protein; Adequate protein and medium dairy (APMD) group: 3–4 dairy servings/day, 15% of their daily energy from high‐quality protein (7.5% of energy as protein from dairy); High protein and high dairy (HPHD) group: 6‐7 dairy servings/day, 30% of their daily energy from high‐quality protein (15% of energy as protein from dairy) Weight loss: < 10% at 4 months (−4.3 ± 0.7 kg)	*n* = 90 premenopausal women Age: 28 ± 1 years (18‐45 years) BMI: 31.5 ± 0.6 kg/m^2^ (27–40 kg/m^2^) T2D: NA – APLD group, *n* = 30 – APMD group, *n* = 30 – HPHD group, *n* = 30	At 4 months APLD group: ↗↗ CTX, ↔ P1NP APMD group: ↗ CTX, ↗ P1NP HPHD group: ↔ CTX, ↗ P1NP HPHD with daily exercise favorably affected BTMs vs. diets with less of these bone supporting nutrients
Brinkworth et al. [22] RCT; Intervention: caloric restriction (~1450–1650 kcal/day) with different dietary composition Duration: 12 months Calcium intake: 802–903 mg/day Vitamin D intake: NA Protein intake: LC 35% vs. LF 24% protein Weight loss: > 10% at 12 months	*n* = 65 adults Age: 51.3 ± 7.1 years BMI: 33.4 ± 4.0 kg/m^2^ (> 30 kg/m^2^) T2D: 0 – Very low‐carbohydrate (LC) diet, *n* = 32 – Higher carbohydrate, low‐fat (LF) diet, *n* = 33	At 12 months (NS between groups) LC group: ↗ CTX LF group: ↗ CTX Weight loss following hypocaloric LC diet compared with LF diet did not differentially affect CTX
Razny et al. [23] RCT; Intervention: caloric restriction (1200 kcal/day for women and 1500 kcal/day for men) ± *n*−3 PUFA (1.8 g/day) Duration: 3 months Calcium intake: no supplements Protein intake: adequate Weight loss: 7% at 3 months	*n* = 64 middle‐aged adults (55% women) Age: 41.0 ± 9.9 years BMI 25–40 kg/m^2^ T2D: 0 – Isocaloric diet with *n*−3 PUFA, *n* = 13 – Isocaloric diet with placebo, *n* = 14 – Low‐calorie diet with *n*−3 PUFA, *n* = 23 – Low‐calorie diet with placebo, *n* = 14	At 3 months Isocaloric ± PUFA: ↔ CTX, ↔ P1NP Low‐calorie diet ± PUFA: ↗ CTX, ↔ P1NP *n*−3 PUFA was without effect on CTX increase
Abilgaard et al. [24] RCT; 25% caloric restriction (first 4 months) and exercise (5 to 6 weekly aerobic training sessions, half of them combined with resistance training) Duration: 12 months Calcium and vitamin D intakes: NA Protein intake: prescription 20% of total dietary intake Weight loss: < 10% at 12 months, −6 kg in the intervention group, −2 kg in the standard care group	*n* = 98 patients with T2D Age: 54 (49–61) years BMI 25–40 kg/m^2^ T2D: 1 – Standard care, *n* = 34 – Lifestyle intervention, *n* = 64	At 12 months Lifestyle intervention: ↗ CTX, ↗ P1NP Standard care: ↔ CTX, ↔ P1NP CTX and P1NP increased significantly more in the lifestyle intervention group compared with standard care

Abbreviations: BTMs: bone turnover markers; CR, caloric restriction; CTX, C‐terminal telopeptide of type I collagen; *n*−3 PUFA, omega‐3 polyunsaturated fatty acid; NA: not available; P1NP, procollagen type 1 *N*‐terminal propeptide; T2D status, 0 – no, 1 – yes; T2D, type 2 diabetes; VLED, very low‐energy diet.

^a^ Data presented as median (interquartile range); ↗: increase; ↘: decrease; ↔: no change.

There is evidence from relatively short‐term weight loss intervention studies, which are typically 3–6 months long, that weight reduction, through energy restriction alone, results in increased BTMs [13, 20, 22, 23]. Most of these studies included women (pre‐ and postmenopausal) and less than 100 participants. In individual studies, P1NP increased modestly (16%–18%), while CTX showed larger increases (ranging from 28% to 101%) [13, 14, 15, 16, 19, 20, 22, 23]. In a meta‐analysis, the increases in P1NP were not statistically significant [26], with these findings collectively suggesting that bone formation may not adequately compensate for increased resorption. This aligns with the well‐established principles that bone formation and resorption are coupled processes, and imbalances when resorption exceeds formation can contribute to net bone loss. The amount of weight loss might be associated with the observed changes in both PINP and CTX [17, 18].

#### 3.1.2 aBMD

In observational studies, weight loss has been consistently associated with bone loss [27, 28, 29, 30, 31] even among those with overweight/obesity who lost weight intentionally [27, 28]. Further reinforcing these epidemiological data, available meta‐analyses of diet‐induced weight loss interventions support small but significant decreases of aBMD at the level of the hip [26, 32, 33] in interventions exceeding 4 months [32] to 6 months [26] (Table 2). These changes correspond to approximately 1%–2% reductions from baseline values and are comparable to the annual hip aBMD losses seen in women during menopause transition [37].

**TABLE 2:** Changes in total hip (TH) and lumbar spine (LS) areal bone mineral density (BMD) in response to weight loss achieved by lifestyle modifications in available meta‐analyses.

Study	Design	TH BMD	LS BMD	Main findings and limitations
Zibellini et al. [26]	P: Healthy adults with overweight/obesity I: Diet‐induced weight loss (alone) – Duration: 3–24 months – Weight loss: 7–11 kg	Diet alone ↓ at 6, 12, or 24 (but not 3) months	Diet alone ↔ at 3, 6, 12 or 24 months	– ↓ TH BMD accompanied by significant ↑ in markers of bone resorption and independent of menopausal status – Inconsistent results on caloric restriction severity (moderate vs. severe) across BMD sites – Analysis based on observational studies and RCTs – Control group present in ~25% of the included studies
Soltani et al. [32]	P: Adults with normal weight/overweight/obesity I: Diet‐induced weight loss ± exercise – Duration: 2–60 months – Weight loss: 0.6–6.9 kg	Diet alone ↓ Exercise alone ↑ Combined diet + exercise ↓	Diet alone ↓ Exercise alone ↔ Combined diet + exercise ↔	– Significant bone loss at the LS and TH in interventions with a duration ≥ 13 months – Exercise‐induced weight loss (minimal weight loss achieved) did not cause bone loss – Population included adults with overweight and obesity, but also adults with a normal weight – ↓ LS BMD was observed only in adults with normal weight
Wright et al. [34]	P: Healthy adults I: Diet‐induced weight loss + protein intake (normal NP or high HP) ± exercise – Duration: 3–18 months – Weight loss ~7 kg	HP vs. NP weightloss diets ↔ loss	HP vs. NP weight loss diets ↓ loss	– Despite attenuating the loss of LS BMD with HP vs. NP weightloss diet, no clinically significant benefit or harm of HP diet on BMD changes during weight loss – Most of the included studies conducted in middle‐aged premenopausal women
Mesinovic et al. [33]	P: Adults with overweight/obesity I: Diet‐induced weight loss ± exercise – Duration: 3–18 months – Weight loss: 2–19 kg (average ~9 kg)	Diet alone vs. combined diet + exercise ↔	Diet alone vs. combined diet + exercise ↔	–No differences in TH bone loss with diet− and diet+ exercise weight loss, no effects on LS – A relatively small number of studies were included in this analysis (*n* = 9)
Yarizadeh et al. [35]	P: Adults with normal weight/overweight/obesity I: Diet‐induced weight loss ± exercise – Duration: 3–18 months – Weight loss: NR	Combined diet + exercise vs. diet alone ↑	Combined diet + exercise vs. diet alone ↔	– Significant ↑ FN and TH BMD (but not LS BMD) with the addition of exercise (effects mainly observed in individuals aged ≥ 65 years and in interventions lasting ≥ 200 days) – Most of the included studies conducted in postmenopausal women
Yazdanpanah et al. [36]	P: Adults with overweight/obesity I: Diet‐induced weight loss ± exercise – Duration: 3–9 months – Weight loss: NR	Combined diet + exercise vs. diet alone ↔	Combined diet + exercise vs. diet alone ↔	– No improvements in TH or LS BMD with exercise (any type) – A relatively small number of studies were included in this analysis of TH BMD (*n* = 4)

*Note*: ↑: increase; ↓: decrease; ↔: no change.

Abbreviations: BMD, bone mineral density; FN, femoral neck; HP, high protein; I, intervention; LS, lumbar spine; NP, normal protein; NR, not reported; P, population; RCT, randomized controlled trial; TH, total hip.

Lumbar spine aBMD appears to be more variably affected, with decreases [38, 39], no changes [16, 40], or even increases [41, 42] reported in original studies. In two meta‐analyses, lumbar spine aBMD remained largely unaffected [26, 33], while in another meta‐analysis, decreases were observed [32] (Table 2). The reasons for these discrepancies are not clear. They may be related to differences in the study design of the included studies in each work (e.g., the meta‐analysis of Soltani et al. [32] included only randomized trials, while the meta‐analysis of Zibellini et al. [26] included observational studies and interventional studies with/without control group).

Artifacts in DXA measurements, particularly in the context of obesity, weight loss, and aging, may also contribute to the heterogeneous aBMD results at the spine after weight loss and the discrepancies in aBMD changes at different skeletal sites [43, 44]. DXA relies on assumptions about soft tissue thickness, which may not hold in individuals with obesity or after significant changes in body composition associated with weight loss, complicating the interpretation of aBMD results. For example, DXA‐derived aBMD reductions at the hip may reflect true bone loss at the cortical and trabecular compartments (see Section 3.2), measurement artifacts, or a combination of both. Nonetheless, these aBMD reductions are supported by few available interventional studies using alternative imaging techniques (computed tomography), which are less affected by soft tissue changes [45]. Conversely, age/disease‐related alterations (e.g., calcifications from atherosclerotic lesions within the aorta, osteophytes, or other degenerative diseases) may blunt the skeletal responses to weight loss, particularly at the spine. This may be one of the reasons why, in sensitivity analyses of the existing meta‐analyses, significant decreases in lumbar spine aBMD were seen only in premenopausal women [26] and younger individuals (age < 65 years) [32]. Similarly, in a post hoc analysis of a weight loss RCT in postmenopausal women with overweight/obesity, vertebral abnormalities (assessed by the criteria for excluding abnormal vertebrae in spine BMD assessment proposed by the International Society of Clinical Densitometry (ISCD)—for a detailed description please refer to the methods of the original work) were present in 44% of the participants at baseline and in 57% of them after weight loss. Notably, moderate caloric restriction was associated with significantly reduced lumbar spine aBMD only after excluding these abnormalities [46].

Many of the available weight loss trials have assessed total body BMD (commonly assessed as part of the evaluation of body composition), which, in current meta‐analyses, remained unchanged [32, 33] or was transiently reduced after diet‐induced weight loss (reductions at 6 months, but not at 3‐ or 12‐month follow‐ups) (Table 1) [26]. The assessment of total body BMD is less clinically relevant as it does not identify specific skeletal sites affected by weight loss. In cases of postmenopausal osteoporosis, bones with a higher proportion of trabecular than cortical bone tissue tend to be more susceptible to deterioration. Hence, when assessing BMD, it is preferable to perform local DXA scans targeting specific bone regions independently or in conjunction with total body bone scans.

#### 3.1.3 Factors That Affect BTM and BMD Changes in Response to Intentional Weight Loss

Several factors can influence changes in BTMs and BMD in response to intentional weight loss in the context of overweight/obesity. Understanding these factors is crucial for assessing the impact of weight loss on bone health and targeting relevant preventive strategies.

##### 3.1.3.1 Population Characteristics (Age, Sex, Menopausal Status, and Presence of Comorbidities)

The effects of weight loss on bone parameters may vary depending on sex, age, menopausal status, and presence of obesity‐related comorbidities, with the potential of more pronounced unfavorable bone effects in individuals who are already at risk of bone fragility. Indeed, significant bone loss at the hip after intentional weight loss is a consistent finding among older adults with overweight/obesity in parallel with significant lean mass loss, but also reductions in fat mass [47, 48]. In contrast, studies in younger individuals have reported more variable results. Similarly, postmenopausal women may experience bone loss even with modest weight loss, while bone loss has been mostly reported after more substantial weight loss in premenopausal women [8]. Despite the findings of individual studies, a meta‐analysis found no differences in bone changes at the hip after diet‐induced weight loss among premenopausal and postmenopausal women [26]. Future studies are needed to directly compare bone responses to weight loss after applying the same weight loss intervention in women with different menopausal status.

The effects of sex on bone changes after weight loss also remain unclear. Most available studies have been conducted either exclusively in women or in mixed populations of women and men, with sex‐specific analyses rarely performed. In the POUNDS LOST trial, diet‐induced weight loss, regardless of the macronutrient composition of the diets, resulted in sex‐specific effects on aBMD at the 2‐year follow‐up. Specifically, women experienced decreases in aBMD at clinically relevant sites after weight loss, while men did not lose bone at the hip and exhibited aBMD increases at the spine (without excluding the possibility of artifacts) [41]. In the Look AHEAD trial, after 1 year of an intensive weight loss intervention, men and women with overweight/obesity and type 2 diabetes (T2D) experienced greater bone loss at the hip, but not at the spine compared to men and women who received diabetes support and education (DSE), without pronounced differences between sexes [49]. In subsequent follow‐ups of the study (4, 8, and 12–16 years), men in the intensive weight loss intervention group experienced a pattern of increased hip bone loss compared to men in the DSE group [50, 51]. In contrast, there were no differences in hip bone loss between women in the two groups during the follow‐up years, although notably, women in the intensive weight loss intervention experienced greater hip bone loss than men receiving the same intervention (8 years: −2.7% vs. −6.5% from baseline; 12–16 years: −2.5% vs. −9.5% from baseline). These results may indicate that over time, other factors become increasingly important for bone loss in women.

Obesity may coexist with other comorbidities including diabetes, metabolic syndrome, and cardiovascular diseases, which may be associated with additional bone impairments (vs. obesity only) and may in turn affect bone responses to weight loss. For example, patients with diabetes present with a low bone turnover, impaired bone quality, and a greater risk of bone fragility compared to individuals without diabetes, with these bone characteristics primarily linked to glucose toxicity with AGEs accumulation, oxidative stress, and chronic inflammation [52]. Given that weight loss in patients with T2D commonly improves glycemic control, such a change could be beneficial for the bone health of these patients. Nevertheless, this hypothesis was not confirmed in the Look AHEAD study, in which weight loss induced by lifestyle changes resulted in improvements in glycemic control but persistent bone loss at the hip in adults with T2D compared to the DSE group [49, 50, 51]. The bone loss reported in this study appears to be comparable to the BMD reductions after weight loss in individuals without diabetes, although no direct comparison was performed.

Notably, there are many other determinants of bone health including personal and/or family history of fracture, use of medications associated with bone loss, untreated thyroid disturbances, and use of antiosteoporotic drugs, hormone replacement therapy, and dietary supplements that may affect bone responses to weight loss and which, however, have not been thoroughly reported in weight loss interventions.

##### 3.1.3.2 Duration of the Intervention

It is important to highlight that to detect changes in aBMD after an intervention, the time gap between the assessments should be sufficiently long to accommodate the process of bone remodeling. Considering that a complete cycle of bone remodeling typically takes 4 to 6 months, the time interval between two measurements in clinical trials should be at least that long to effectively identify changes in aBMD [26]. Given that most studies in the field have not been designed to assess bone changes as primary outcomes, they often have a duration of 3 to 6 months, with more recent studies providing aBMD assessments after longer follow‐ups (> 12 months). Some studies suggest that weight loss‐induced bone loss is not temporary but persists for months or even years after the end of a single/given weight loss intervention, highlighting possible long‐term risks associated with repeated weight loss diets on bone health [46, 50, 51, 53]. Although findings of studies with longer follow‐ups provide clinically meaningful BMD measurements, they should be interpreted after considering relevant confounding factors including the practices of dieters (e.g., healthier eating habits or return to unhealthier dietary habits, exercise, or sedentary lifestyle) and weight loss/maintenance/regain after the end of the active intervention (see Section 3.1.3.6).

##### 3.1.3.3 Amount and Pace of Weight Loss

Although the threshold effects of 5% weight loss are generally accepted for metabolic benefits, there is no clearly established weight loss cutoff over which bone loss occurs. It has been proposed that ≤ 5% weight loss has no/little effect on BMD, while weight loss exceeding 10%‐15% of initial body weight may result in more pronounced decreases in BMD [8]. These effects appear to depend on the population under investigation (see Section 3.1.3.1), with significant bone loss commonly observed after subtle weight reduction in postmenopausal women and older adults.

The pace of weight loss can have various effects on health outcomes; nevertheless, the impact of this parameter on BMD remains poorly investigated and often confounded by other weight loss aspects (i.e., amount of weight loss). For example, rapid weight loss as a result of severe energy restrictions derails bone remodeling, favoring bone resorption and lowering BMD [54]; nevertheless, it remains unknown whether or not the same amount of weight loss achieved over a more extended period moderates these unfavorable bone effects.

##### 3.1.3.4 Degree of Dietary Energy Restriction and Dietary Composition

###### 3.1.3.4.1 Moderate Energy Restrictions (MERs) vs. Very Low‐Energy Diets (VLEDs)

In theory, VLEDs (dietary prescriptions ≤ 800 kcal/day) are expected to have more pronounced unfavorable effects on bone than MERs (reductions in caloric intake by 500–1000 kcal/day) due to the drastic reductions in energy intake and potential nutrient deficiencies (unless they are based on commercial products which are commonly nutritionally replete). However, a sensitivity analysis in the meta‐analysis of Zibellini et al. suggested that VLEDs or low‐energy diets (LEDs) did not pose a greater risk to bone health than MERs, despite the greater weight loss achieved with VLEDs and LEDs (−11.1 kg vs. −9.6 kg) [26]. Specifically, VLEDs/LEDs and MERs affected different skeletal sites, with hip BMD declining after MERs and spine BMD after VLEDs/LEDs. In contrast, in a subsequent RCT by Seimon et al. that provided direct comparisons of MERs with VLEDs at 1‐year follow‐up, postmenopausal women who received the VLED experienced greater weight loss (−15.3 kg vs. −8.4 kg) and an approximately 2.5‐fold greater loss of total hip aBMD (−3.3%) compared to those randomized to MER, with this difference remaining significant after accounting for weight loss differences [54]. Lumbar spine and total body aBMD also decreased at 12 months after both diets, with no differences between the two diet groups. Notably, the aBMD reductions at the level of the hip occurred despite a dietary protein prescription of 1 g/kg of actual body weight/day in both groups and even though the total meal replacement products (VLED) were meeting national nutritional requirements for calcium and vitamin D. Further studies are needed to confirm these findings and further explore if VLEDs have differential effect on BMD depending on whether they are food‐based or rely on replacement products.

###### 3.1.3.4.2 Ketogenic Diets/Low‐Carb Diets

The effects of ketogenic diets (i.e., high in fat and low in carbohydrates) on bone health are a topic of ongoing research and debate. Some preclinical and clinical studies suggest that a ketogenic diet might negatively affect bone health [55, 56]. However, most human studies exploring the effects of these regimes have been conducted in children who follow ketogenic diets as part of their treatment for neurological conditions. Among populations with overweight/obesity and related comorbidities, a few studies have found no adverse effects of low‐carbohydrate diets on BTMs or aBMD compared to other weight loss diets [22, 57, 58] although in some of them aBMD reductions from baseline were observed independently of diet composition during the follow‐up [22]. These differential bone effects in these different populations may be related to the stricter carbohydrate restrictions and specific diet formulas prescribed in children with neurological conditions, the complex interactions of diet with medications and comorbidities, and the potentially more pronounced effects of nutritional interventions on the growing skeleton [56]. Given that ketogenic diets lead to metabolic adaptations (e.g., ketosis) that could influence bone health and may be insufficient in nutrients important for bone health, such as vitamin D and calcium (unless supplemented), the effects of their long‐term and repeated use in individuals with overweight/obesity require further investigation.

##### 3.1.3.5 Meal Timing

In addition to what is eaten, when eating is taking place is also important. Emerging research indicates that intermittent fasting (IF‐approaches that emphasize the timing of eating rather than diet quantity or quality) can be an alternative for weight loss [59]. Recent research suggests that time‐restricted eating and alternate‐day fasting practiced for up to 6 months resulted in modest weight loss (< 5% of the initial body weight) and had no adverse effects on BTMs or total body BMD [60]. Among the different IF approaches, time‐restricted eating with an eating window of 8–12 h/day may even have small bone‐sparing effects in the context of weight loss as suggested by smaller reductions in the bone formation marker P1NP [61], no increase in the resorption marker CTX [62], and unchanged or increased total body bone mineral content (BMC) (vs. reductions in the control groups) [61, 62]. Interestingly, most IF approaches have resulted in less and often nonclinically significant weight loss compared to conventional weight loss diets, had a short duration, and only assessed total body BMC/BMD. Thus, further research is required to better characterize bone changes at the level of the hip and lumbar spine in response to various IF approaches practiced for longer periods (i.e., > 6 months) and compare them to other weight loss approaches to clarify the clinical significance of the findings of the few available studies.

##### 3.1.3.6 Weight Regain

Several studies support that BTM imbalances [13, 14, 16, 18, 20] and bone loss [14, 53, 63, 64] after a weight loss attempt endure over time and persist even after weight loss reaches a plateau or is regained. For example, in the TEMPO study, significant reductions in total hip aBMD were mainly observed in the first year of the weight loss intervention and in parallel with weight loss (moderate weight loss group: −8.8%, severe weight loss group: −17.3%) [54]. At the 36‐month follow‐up, despite significant weight regains (moderate weight loss group: +3.5 kg, severe weight loss group: +7.3 kg), no improvements in total hip aBMD were observed, with significant declines between baseline and 36 months [53]. These findings suggest enduring effects of weight loss on bone health and may be linked to unfavorable metabolic effects of weight regain, i.e., weight regain is commonly achieved by regain and redistribution of fat mass and increases in (pro)inflammatory cytokines, which can negatively affect bone health.

Some other studies support some mitigating effects of weight regain on bone loss due to a single weight loss attempt. Von Thun et al. showed that postmenopausal women with overweight/obesity who experienced approximately 10% weight loss and regained approximately 70% of it over 18 months experienced less trochanter and 1/3 radius BMD loss compared with those who maintained a reduced body weight [64]. In the Look AHEAD study, the group who followed an intensive lifestyle weight loss intervention regained some of the weight lost between the first year and the final visit at 12 to 16 years [51]. The intervention group (vs. the DSE group) experienced greater bone loss after 1 year, but the difference between the treatment groups did not persist in the long term (12–16 years).

Collectively, these findings have implications for individuals who are involved in repeated cycles of weight loss and regain (yo‐yo dieting) and who may experience cumulative adverse effects on bone health, as their bones may not fully recover from prior losses. Further studies are needed to explore the effects of repeated weight loss efforts on aBMD and identify factors/practices that may be related to exacerbated bone loss or aid bone recovery after weight loss.

### 3.2 The Effects of Intentional Weight Loss on Bone Microstructure

Although variations in soft tissue thickness in studies of longitudinal weight change also influence the accuracy of volumetric BMD (vBMD) assessed by high‐resolution peripheral quantitative computed tomography (HR‐pQCT) [65], several cohort studies have indicated negative associations between weight loss and bone microarchitecture and strength in older individuals. Trabecular microarchitecture appears to be impaired by weight loss, although current evidence on this aspect is less consistent. In the AGES‐Reykjavik Study, bone strength loss assessed from volumetric QCT images of the proximal femur increased in women with higher degrees of weight loss [66]. In the Osteoporotic Fractures in Men (MrOs) study, community‐dwelling older men with accelerated bone loss experienced greater weight loss than other men [67]. Weight loss was associated with detrimental effects on bone strength and total and cortical vBMD at distal skeletal sites, as well as detrimental effects on cortical thickness at distal and proximal sites. However, weight gain late in life is not associated with a commensurate increase in bone strength [68]. In 70‐year‐old women and men in the Framingham Offspring Cohort, both recent and long‐term weight loss was negatively associated with cortical and trabecular vBMD and microarchitecture in the weight‐bearing skeleton [69]. In the SWAN Longitudinal HR‐pQCT Study, a longitudinal study in postmenopausal Black and White postmenopausal women, those who lost weight over the follow‐up period had higher rates of bone loss at the peripheral skeleton, particularly at the tibia, than those who maintained or gained weight [31].

### 3.3 The Effects of Intentional Weight Loss on Fracture Risk

In observational studies, weight loss has been consistently associated with increased fracture risk [27, 70, 71, 72, 73, 74], with only a few studies having evaluated the underlying reasons for weight loss [27, 70, 71] and stratified analyses by overweight/obesity status [27]. These are important considerations because unintentional weight loss may result from illness, which can affect bone loss and the risk of falls and fractures independently. Conversely, intentional weight loss is commonly achieved by lifestyle changes, some of which (i.e., exercise) may have protective bone effects. Furthermore, obesity is known to influence susceptibility to fractures mainly through a higher BMD (due to greater skeletal loading), greater impact forces in case of falling, and protective effects of soft tissue padding [6, 75]. Limited data have demonstrated a link between intentional weight loss and increased fracture risk at different anatomical sites. In a post‐hoc analysis of the Women's Health Initiative (WHI) in postmenopausal women aged 50–79 years, intentional weight loss was associated with a higher incidence of lower limb fractures, but a lower incidence of hip fractures over a mean follow‐up of 11 years [70]. Among older women with overweight/obesity, intentional weight loss (≥ 5% from baseline) was linked to a significantly increased risk of hip fracture [27]. Similarly, in a large cohort of older men, moderate weight loss (≥ 10%) was associated with a 1.6‐fold higher adjusted risk of clinical fracture at the hip, spine, or pelvis [71]. Although the observed associations vary to some extent according to the study design and population characteristics (e.g., age, sex, BMI, health status), these findings collectively suggest that voluntary weight loss may increase fracture risk in populations already at risk, such as postmenopausal women and older adults.

Using data from interventional studies, a meta‐analysis showed that lifestyle weight loss programs were not associated with an increased risk of any type of fracture [76]; however, the included studies had several limitations and varied considerably in design, making it challenging to draw conclusions. This is because most studies reported fracture data as adverse events rather than as a priori outcomes, had small sample sizes and short follow‐ups, and were conducted in heterogeneous populations ranging from relatively young individuals to patients with specific obesity‐related comorbidities, such as T2D. Notably, the Look AHEAD trial in patients with T2D aged 45–76 years (*n* = 5145) showed that 6%–9% weight loss achieved by lifestyle modifications (caloric restrictions and physical activity) and some of it maintained over a decade was associated with a 39% greater risk of fragility fractures, defined as fractures at the hip, pelvis, and shoulder [77]. Further large randomized trials with similar long follow‐ups allowing for unveiling any potential BMD changes and fracture events are needed to confirm and extend these findings in individuals with obesity with/without T2D/other comorbidities.

Bone loss as a result of a single weight loss attempt may persist over time after a weight loss plateau or regain, implying that individuals engaged in repeated weight loss attempts may be at a higher risk of fracture. Indeed, observational studies support unfavorable associations between weight variability [78, 79], self‐reported weight cycling [80, 81], and the incidence of fractures. In one of the first studies addressing this question, those in the highest quartile of weight variability (defined as the root mean square around the slope of weight) were found to be at a higher risk of total and hip fractures than those in the lowest quartile [78]. Further studies have extended these findings to weight cycling by showing a greater risk of forearm fractures in men [80] and nonvertebral fractures in women [81] among those who recalled repeated weight loss episodes (≥ 4 in men and ≥ 11 in women). In contrast, in a 4‐year follow‐up of the Look AHEAD trial that included participants who received lifestyle intervention and experienced weight loss during the first year, patterns of weight change (continued weight loss or weight maintenance, weight regain, and weight cycling) in the following 3 years were not associated with incident fractures [82]. Several reasons may explain the discrepancy in the results, including differences in the assessment of weight changes (measured vs. self‐reported), definitions of weight cycling, and the number of weight cycling episodes.

### 3.4 Mechanisms of Bone Loss During Lifestyle‐Induced Weight Loss

**FIGURE 1:**
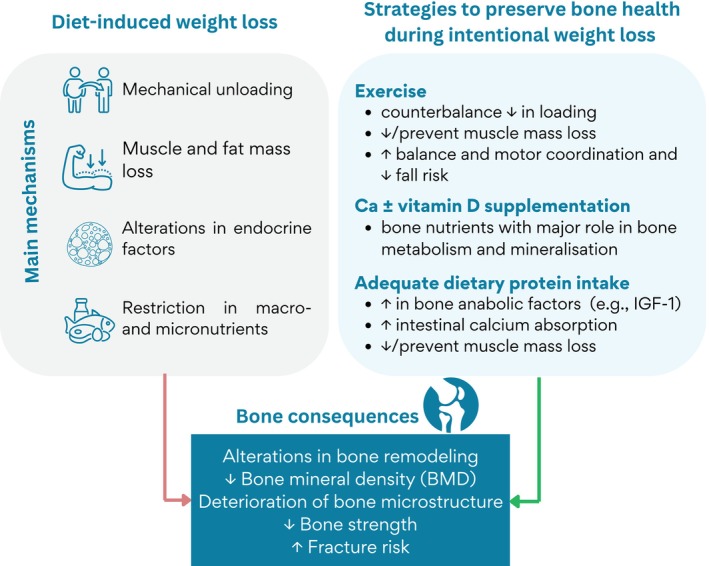
Summary of the negative bone consequences of diet‐induced weight loss and strategies to preserve bone health in the context of intentional weight loss. Ca, calcium; IGF‐1, insulin‐like growth factor‐1. [Color figure can be viewed at wileyonlinelibrary.com]

To effectively develop and assess strategies aimed at mitigating or even preventing bone loss associated with weight loss, it is important to understand the mechanisms that mediate these effects. We briefly summarize the potential mechanisms before discussing the relevant interventions that can target these mechanisms with the goal to reduce bone loss following intentional weight loss (Figure 1).

Bone can adapt its mass, structure, and strength according to the load, which can be the result of physical activity or increased body weight, while mechanical unloading is identified as one of the main mediators of the effects of weight loss on bone [83, 84]. Sclerostin, a protein secreted by osteocytes that senses changes in mechanical loading, is elevated after diet‐induced weight loss and is linked to decreased bone formation through inhibition of the canonical Wnt signaling pathway, a critical pathway for the differentiation, proliferation, and activity of osteoblasts [85]. The loss of lean and fat mass (in absolute terms) that occurs during weight loss may negatively affect bone health by contributing to the unloading of the skeleton. In addition to the mechanical connection of bone with muscle and fat, these tissues are further linked biochemically. This communication involves cytokines derived from myocytes (myokines), adipocytes (adipokines), and bone cells (osteokines), as well as systemic endocrine factors (sex steroids, growth hormone, and insulin‐like growth factor‐1 (IGF‐1)) with actions on bone, muscle, and adipose tissue [86, 87]. From a nutritional perspective, hypocaloric diets may provide lower amounts of nutrients important for bone health, including protein, calcium, and vitamin D. Deficiencies in these nutrients may initiate and propagate catabolic procedures, including secondary hyperparathyroidism and associated bone loss or muscle wasting [88, 89].

On the other hand, weight loss can lead to reductions in chronic inflammation (due to obesity), which can be beneficial for bone health [90, 91, 92]. Chronic inflammation through the secretion of inflammatory cytokines and modification of immune responses is known to affect bone metabolism, by promoting bone resorption and inhibiting bone formation. The relationship between weight loss, inflammation, and bone health is complex and can be affected by many factors, including the amount and type of weight loss (fat vs. muscle), an individual's diet and nutritional status, the presence of other health conditions, and the specific methods used to achieve weight loss.

Bone compartments may be differentially affected by these mechanisms associated with weight loss. Trabecular bone, with its higher surface area and metabolic activity, is generally more responsive to hormonal and metabolic changes than cortical bone, although the latter can also be affected. Cortical bone is more influenced by mechanical loading. The hip, which contains both trabecular and cortical bone, may therefore be particularly susceptible to the combined effects of mechanical unloading and systemic changes, potentially explaining why reductions in hip BMD are more consistently observed following weight loss.

### 3.5 Strategies to Preserve Bone Health During Intentional Weight Loss

#### 3.5.1 Exercise

The addition of exercise to weight loss programs increases mechanical strain and can partially counterbalance the decline in loading due to weight loss. Resistance exercise alone or combined with aerobic exercise, but not aerobic exercise alone, has been associated with less pronounced weight loss–induced increases in BTMs compared to patients on caloric restrictions only [16, 19]. Two recent meta‐analyses concluded that the addition of exercise attenuates to some extent the BMD reductions seen during diet‐induced weight loss [33, 35], while another one supported some skeletal benefits on total body BMD for resistance exercise only [36] (Table 2). Various factors have been identified as limiting the magnitude of the beneficial effects of exercise on bone in these meta‐analyses including the limited duration of exercise interventions (3–6 months), lack of supervision/compliance during exercise training, and attenuated anabolic responses to exercise in older adults. Further extending these results, adding resistance exercise to a weight loss diet may attenuate long‐term (30‐month follow‐up) bone loss at the level of the hip (vs. diet‐induced weight loss) and increase lumbar spine BMD [45]. Some recent data also suggest that structured exercise programs during weight loss may be beneficial in individuals with specific obesity‐related comorbidities. In the U‐TURN trial, patients with T2D were randomized to an intensive lifestyle intervention group (aerobic and resistance exercise training along with 25% caloric restriction) and a standard care group. The intensive lifestyle intervention group experienced greater weight loss and significant increases in BTMs over the 12‐month follow‐up compared to the group who received standard care. Taking into consideration that individuals with T2D often present with low BTM levels, this increase in BTMs along with a preservation in BMD was suggested by the authors to reflect improvements in bone health following weight loss in this population [24].

Overall, resistance exercise is the type of exercise that provides the greatest osteogenic stimuli, while it can also help preserve muscle mass during weight loss. Preventing falls is also important for reducing fracture risk in individuals with overweight/obesity. Towards this goal, balance training has been shown to improve balance and motor coordination and lower fall risk [93]. Aerobic exercise has a lower impact on bones (i.e., less osteogenic); nevertheless, it is related to better cardiovascular fitness, which can indirectly benefit bone health. Some studies have highlighted the importance of exercise for weight loss and bone health when other weight management treatments are used. For example, a recent study indicated that the combination therapy of liraglutide and exercise resulted in the most substantial weight loss and preservation of hip and spine BMD (vs. less weight loss and bone loss in patients who received liraglutide alone) [94]. Similarly, exercise training as part of post‐bariatric surgery care improved weight loss, body composition [95] and attenuated bone loss [95, 96].

There is an ongoing interest in finding alternative load‐based approaches for mitigating bone loss during weight loss. Weighted vests may allow an individual to replace the lost weight externally, and preliminary data support that their use can enhance muscle and bone outcomes during weight loss [97]. If proven beneficial in larger, longer‐term studies, such an intervention could be useful for people who comply poorly with exercise or who have limitations in performing certain exercise routines.

#### 3.5.2 Adequate Dietary Protein Intake

Dietary protein is probably the most studied macronutrient in relation to bone, with well‐established anabolic effects, including increases in circulating IGF‐1 levels, enhanced intestinal calcium absorption, and maintenance of muscle mass [98]. In the context of diet‐induced weight loss, a higher protein intake is also thought to be beneficial for bone health. For example, older adults who followed a hypocaloric, nutritionally complete, high‐protein (≥ 1 g/kg body weight/day) diet for 6 months lost approximately 9% of their initial body weight but had similar aBMD (total hip, femoral neck, and lumbar spine) to controls who maintained their body weight over the same period [99]. In contrast, a meta‐analysis of studies that compared differences between weight loss diets with high and normal protein content during weight loss showed that protein quantity had only modest effects on bone changes, with little/limited clinical significance [34]. These findings may, in part, reflect differences in study demographics and baseline dietary characteristics or may be explained by other factors related to protein intake, which were not considered in this meta‐analysis. Indeed, in addition to protein quantity, protein quality and complex interactions of food matrices may also be important for bone outcomes and require further investigation.

#### 3.5.3 Key Bone‐Related Micronutrients via Diet or Supplementation

##### 3.5.3.1 Calcium

During diet‐induced weight loss, calcium supplementation of 1 g/day was shown to slightly improve lumbar BMD and significantly reduce PTH levels [100, 101]. Higher doses of calcium supplementation (1.7 g/day), combined with adequate vitamin D status, attenuate but do not fully prevent bone loss in postmenopausal women losing body weight [102]. Additionally, calcium supplementation of 1.8 g/day has been strongly associated with increased femoral neck BMD and elevated serum levels of 25‐hydroxyvitamin D (25[OH]D) in premenopausal women with overweight during weight loss [103].

A food‐first approach to calcium intake is generally recommended, not only due to concerns about excessive calcium supplementation, especially in the context of high total calcium intake (i.e., gastrointestinal distress, nephrolithiasis, and adverse cardiovascular events) [104, 105] but also because calcium‐rich foods, particularly dairy, provide additional beneficial nutrients within a complex food matrix [106]. Few further studies have examined whether calcium sources (supplements vs. dairy products) have differential effects on bone outcomes in the context of diet‐induced weight loss. An RCT comparing two calcium sources to a placebo reported better bone outcomes with calcium and vitamin D supplements, while dairy products were linked to improved metabolic outcomes, including a lower decrease in lean mass and greater reductions in fat mass [107]. Another study comparing the skeletal responses to different calcium sources and weight loss found lower urinary markers of bone resorption with calcium lactate supplementation, an effect not observed with calcium phosphate or milk [108].

It remains unclear why some of the benefits seen with calcium supplementation on bone during weight loss have not been reported with dairy foods. Dairy products are a major source of calcium, but also supply several other nutrients including high‐quality proteins, potassium, magnesium, vitamin D (if fortified), and certain B vitamins. Current literature supports that dairy foods exert health benefits (including skeletal benefits) that arise not only from their individual components but also from the synergistic interactions between these components within the unique structure of dairy foods (i.e., the dairy matrix health effects) [106]. Supporting the benefits of dairy products, evidence suggests that diets higher in dairy products help mitigate weight loss‐induced bone loss and reduce bone resorption markers during diet‐induced weight loss [21, 109, 110].

No data are available on the bone effects of different dairy products, such as yogurts, which can serve as a source of probiotics and may support favorable gut‐bone axis interactions [106, 111]. There are also no data on whether the fat content of dairy products is relevant for bone health in the context of weight loss. For decades, dietary guidelines have favored low‐fat dairy products for the general population, including individuals aiming to lose weight mainly due to concerns about saturated fat and cardiovascular disease risk. Despite their high saturated fat content, recent literature suggests that certain dairy foods, and especially yogurt and cheese, may have a more nuanced impact on health, potentially due to their unique dairy matrices. Future research is needed to explore how different types of dairy foods affect bone health during weight loss.

#### 3.5.4 Vitamin D

Vitamin D metabolism is altered in obesity, often leading to lower circulating levels of 25(OH)D. These reductions are attributed to several factors, including insufficient sunlight exposure, reduced hepatic synthesis of vitamin D, and its volumetric dilution in excessive adipose tissue [112]. Levels of 25(OH)D are inversely correlated with BMI and fat mass [113, 114, 115]. Conversely, weight loss has been associated with increased serum 25(OH)D levels in women with overweight or obesity [116, 117], supporting the hypothesis that vitamin D is released from adipose tissue during fat loss.

A limited number of studies have examined the effects of vitamin D3 supplementation on bone parameters during weight loss. For example, an RCT by Mason et al. found no benefit of vitamin D supplementation compared to placebo in preventing lumbar or femoral bone loss in postmenopausal women with obesity after 1 year of a weight loss intervention [118]. Another RCT compared three doses of vitamin D3 (600/2000/4000 IU/day) with calcium (1200 mg/day) in postmenopausal women with overweight and obesity [119]. After 1 year of supplementation, the three experimental groups experienced modest but comparable weight loss and increases in their 25(OH)D concentrations in a dose‐dependent manner. No differences were observed in BTMs or BMD between groups; nevertheless, the group receiving the higher vitamin D3 doses experienced increases in the cortical thickness of the tibia compared to mild decreases seen in the group receiving the lowest vitamin D3 dose.

## 4 Conclusions and Future Directions

Weight loss achieved through lifestyle modifications results in a 5%‐10% weight loss and increases in bone resorption with insufficient formation and is associated with relatively small but persistent reductions in aBMD at clinically relevant sites. These aBMD reductions appear to be more consistent at the hip, with more variable findings at the spine. It remains, however, unclear whether such differences reflect true site‐specific effects of weight loss or are influenced by artifacts in DXA measurements, particularly in the context of obesity, weight loss, and aging. Based on limited available data, intentional weight loss may increase fracture risk at specific skeletal sites, particularly when other risk factors are present. Current evidence supports that promoting regular exercise, ensuring adequate calcium intake through diet and/or supplements, maintaining sufficient vitamin D levels through supplementation, and consuming higher amounts of dietary protein can support bone health, although they may not entirely offset the unfavorable skeletal effects associated with intentional weight loss.

Further research is needed (Box 1) to better understand the skeletal effects of weight loss interventions that reflect real‐world clinical scenarios. This includes exploring the impact of repeated weight loss and regain (weight cycling), as this is common in clinical and community settings and may have cumulative effects on bone health. Studies comparing different rates of weight loss, such as gradual versus rapid reductions, and varying dietary approaches (e.g., low‐carbohydrate, high‐protein/carnivore, plant‐based, meal timing strategies) are also needed to determine whether some methods are more detrimental or protective for the skeleton. Importantly, research should aim to identify individuals who may be more susceptible to the adverse skeletal effects of weight loss, such as postmenopausal women, older adults, or those with poor musculoskeletal health at baseline. Additionally, it is important to further clarify and confirm whether observed changes in BMD or microstructure or other factors (e.g., changes in muscle strength or fall frequency/patterns) following weight loss ultimately translate into an increased risk of fractures. Finally, evaluating the effectiveness of novel or combined strategies such as exercise, nutritional support, or pharmacological interventions in preserving bone health during weight loss could help inform more effective and individualized approaches to obesity treatment.

BOX 1 **Directions for Future Research.**

**Prospective Studies**

- Take into account whether the weight loss was intentional or unintentional, and stratify the analyses according to participants’ body weight status (normal weight, overweight, obesity).
- Assess weight status and body composition parameters at multiple time points.
- Include long‐term follow‐ups to allow the detection of hard outcomes (e.g., incidence of fractures and falls).
- Identify the onset and magnitude of weight loss that may be more consistently associated with unfavorable bone outcomes.
- Address/consider important confounding factors including demographics, comorbidities and medication use and lifestyle factors dietary composition, micronutrient intakes and physical activity.

**RCTs Should**

- Have well‐designed methodologies, sufficient numbers of participants to detect meaningful changes/differences in bone outcomes, and longer durations.
- Detail compliance and challenges related to weight loss interventions.
- Investigate the dose‐response relationships of weight loss and bone changes.
- Differentiate the effects of rapid weight loss vs. the effects of the same amount of weight loss achieved gradually (at a slower pace).
- By using the same weight loss regime, compare its bone effects in different populations.
- Explore the impact of repeated weight loss and regain (weight cycling).
- Explore and compare different dietary approaches commonly used for weight loss (e.g., low‐carbohydrate, high‐protein/carnivore, plant‐based, meal timing strategies).
- Clarify whether changes in BMD, bone microstructure, or related factors after weight loss lead to increased fracture risk.
- Explore further/novel ways to prevent bone loss (weighted vests, meal timing, and types of exercise).
- Elucidate mechanistic pathways.

## Conflicts of Interest

M.A.L. reports occasional consulting for BMS. J.M.L. has occasionally provided expert interventions for Amgen, Binc, ISA, Lilly, Novo Nordisk, and Link‐Up and is a member of scientific boards for Aprifel, Bel, FICT, Holder, and IOT. R.C. has received support or honoraria from Abbvie, Amgen, UCB, Lilly, Mereo, Alexion, Amolyt, Kyowa‐Kirin, Pfizer, Alfasigma, Medac, Nordic, Theramex, Novartis, Viatris, and Fresenius‐Kabi. B.C. has occasionally participated as an expert or speaker for Alexion, Amgen, Aptissen, Expanscience, Lilly, Kyowa‐Kirin, Novartis, Theramex, UCB, and Viatris. J.P., T.T., E.B., and M.P. declare no conflicts of interest.

## Data Availability

Data sharing is not applicable to this article as no new data were created or analyzed in this study.
